# An ethnopharmacological survey of herbal medicines use among pregnant and postpartum women in Souss Massa (Morocco)

**DOI:** 10.3389/fphar.2025.1567930

**Published:** 2025-05-09

**Authors:** Afaf Bouqoufi, Laila Lahlou, Fatima Ait El Hadj, Said Boujraf, Mohammed Abdessadek, Youssef Khabbal

**Affiliations:** ^1^ Laboratory of Innovation Research in Health Sciences, Team of Therapeutic Innovation, Translational Research and Epidemiology, Faculty of Medicine and Pharmacy Ibn Zohr University, Agadir, Morocco; ^2^ Medical and Clinical Pharmacology Department, University Hospital Center Sous Massa, Agadir, Morocco; ^3^ Clinical Neurosciences Laboratory, Faculty of Medicine, Sidi Mohamed Ben Abdellah University, Fez, Morocco; ^4^ Laboratory of Analysis, Modeling, Engineering, Natural Substances and Environment, Polydisciplinary Faculty of Taroudant, Ibn-Zohr University, Agadir, Morocco

**Keywords:** traditional medicine, herbal medicine, childbirth, pregnancy, ethnopharmacology, survey

## Abstract

**Introduction:**

Pregnant women use medicinal plants for the treatment of illnesses associated with pregnancy. Women might resort to using medicinal plants because they are easily accessible and inexpensive. This study aims to determine the prevalence, to document and to analyse the traditional use of medicinal plants during pregnancy.

**Methods:**

A cross-sectional study was conducted among pregnant and postpartum women attending the Obstetrics and Gynecology Service at the regional hospital center, Hassan II of Souss Massa region. An Ethnopharmacological survey was used for data collection. After collection, data were coded, entered, and analyzed by Jamovi Software.

**Results:**

A total of 420 women participated in this study. The mean age was 28.7 ± 6.35. The prevalence of herbal medicine consumption among pregnant and postpartum women is 48% with CI 95% (43.2%–52.7%). 35 varieties of medicinal plants from 22 families were used during pregnancy. Each of the other families had one species. Plant extracts were primarily prepared through decoction, infusion, maceration, Fumigation, extraction, and powder. The oral route was the most common route of administration used, about the number of plates used by pregnant and postpartum women (90.9%).

**Conclusion:**

The utilization of herbal medicine among pregnant mothers in this study was high in the region of Souss Massa. This research expands our understanding of the role that different plant species have in the management of disorders that affect women during pregnancy.

## Introduction

Herbal medicine is known as the practice of using plant-derived substances or preparations for the treatment, diagnosis, prevention, and maintenance of health (World Health Organization, 2001). Based on the World Health Organization’s data, up to 80% of Africans still depend on traditional medicine to manage their healthcare requirements, while in China, traditional medicines make up an estimated 40% of all healthcare services (World Health Organization, 2002). Throughout the world, herbal medicines use is frequently used during pregnancy (Adams et al., 2009; Hall et al., 2011; Pallivalappila et al., 2013). Based on a study reported that approximately of 50% of pregnant women from various non-American nations utilized at least one herbal medicine (Strouss et al., 2014). In the Arabian countries, a systematic review investigated the prevalence of using herbal medicine in pregnancy in the Middle East and North Africa showed a high prevalence varied from 22.3% to 82.3% (John and Shantakumari, 2015). In southern Morocco, a cross-sectional study conducted by Kamel et al. (Kamel et al., 2022) found that 375 pregnant women out of 560 (70%) used herbal products during pregnancy and these were used for a variety of things, including pain relief, easing childbirth, preventing flu syndrome, treating anemia, and inducing delivery (Kamel et al., 2022).

Throughout pregnancy, the female’s body experiences physical and physiological changes which can cause a variety of disorders related to pregnancy, such as heartburn, nausea, vomiting, and constipation (Bruno et al., 2018). Pregnancy is mostly accompanied by minor complaints. However, these complaints can rarely cause serious problems that could affect the health of the mother and newborn and eventually result in their mortality. The World Health Organization and the United Nations International Children’s Emergency Fund reported that each year, health problems related to pregnancy or childbirth cause the deaths of more than 500,000 women and four million newborns who are less than 1 month old (La Situation Des Enfants Dans Le Monde, 2009; World Health Organization, 2007). Poverty and risky pregnancies (refer to pregnancies in which the health of the mother, the baby, or both is at an increased risk of complications before, during, or after childbirth) are closely related to 99% of all deaths worldwide as represented in Asia and Sub-Saharan Africa (World Health Organization, 2008). Furthermore, the use of conventional medication during pregnancy has been greatly limited due to the first-term pregnancy’s teratogenic sensitivity and the adverse effects caused by synthetic medicines, for example *Aristolochia spp*. (Birthwort) contains aristolochic acids, which are known to cause severe kidney damage, increase the risk of urothelial cancer, and lead to DNA mutations. Despite its historical use in traditional medicine, Aristolochia has been banned in many countries due to its carcinogenic properties. Another highly toxic herb is *Aconitum spp.* (Aconite, Monkshood, Wolf’s Bane), which contains aconitine, a potent neurotoxin and cardiotoxin. Even in small doses, aconite can cause severe cardiac arrhythmias, paralysis, and respiratory failure, often leading to death. Due to their concerns for the fetus’ safety, pregnant women frequently turn to natural medicin rather than prescribed medications to deal with these changes. To resolve some of these problems pregnant women favor the return to phytotherapy (Pinn and Pallett, 2002). Previous studies have reported the use of herbal medicine among pregnant women according to the geographical location, socio-cultural character, and ethnicity of the pregnant women, the prevalence of herbal medicine used to treat maternal problems ranged widely from 33% to 77.1% (Abd El-Mawla, 2020; Aljofan and Alkhamaiseh, 2020; Eid et al., 2020; Sadeghia and Mahmood, 2014).

The limited clinical data on the safety and effectiveness of herbal medicines makes the benefit-risk assessments very difficult but some herbs have been identified as being harmful and contain toxic elements (Ahmed et al., 2017). Furthermore, nothing is known about the interactions between different herbs, drugs, and foods. Due to additives or contamination with poisonous metals or even hidden conventional medicines, herbal treatments have been associated with undesirable side effects (Saper et al., 2004). The risk of undesired effects is increased by a limited understanding of the potential risks of some herbs during pregnancy as well as the fact that natural herbs and vitamin supplements are not subject to the Food Drugs Administration assessment procedure necessary for prescribed drugs (Skerrett and Harvard Health, 2012).

Morocco is a Mediterranean nation that has a rich heritage of phytotherapy (Jouad et al., 2001). Many authors have investigated traditional pharmacopeia in various regions of Morocco. There is a lack of available information on the usage of herbs in pregnancy, especially in the Souss Massa region. However, some studies reported the use of herbal medicines among Moroccan Southern populations (El-Ghazouani et al., 2021). The Agadir Ida Outanane province is particularly notable for its rich diversity of medicinal plants. (*Thymus vulgaris L*., *Lavandula angustifolia* Mill., *Argania Spinosa* (L). However, this region is characterized by unregulated and excessive use of botanical species, surpassing the natural regeneration capacity and posing a threat to certain species (Ouhaddou et al., 2014).

This study aims to determine the prevalence, and focus on documenting and analyzing the traditional use of medicinal plants consumption during pregnancy, labor, and after delivery in the Hospital Center Hassan II at Souss Massa region.

## Material and methods

### Study area

**FIGURE 1:**
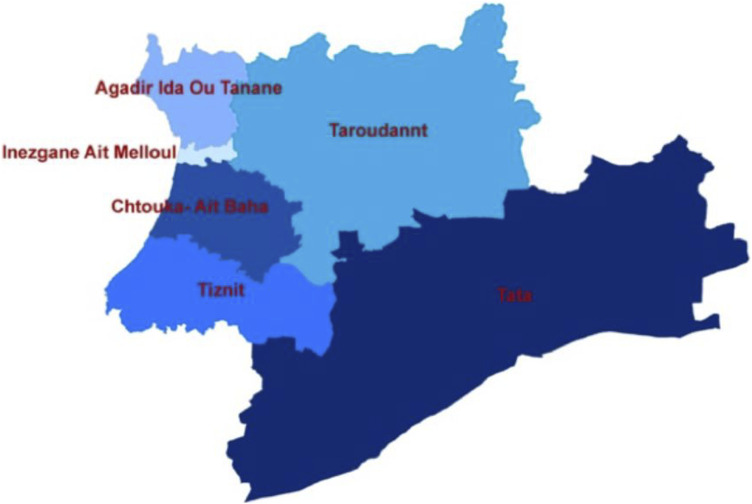
Map of the Sous Massa Region and boundaries of the study area; High Commission for Planning–Souss Massa regional directorate.

Souss Massa region Covering an area of 53,789 km^2^, i.e., 8% of the national territory, the Souss Massa region is made up of 2 prefectures: Agadir Ida Outanane and Inezgane Ait Melloul and 4 provinces: Chtouka Ait Baha, Tiznit, Taroudannt, and Tata. The total population reached 2 million 677 thousand inhabitants in 2014, which is 8% of the national population. The rate of urbanization of the regional population reached nearly 56% (compared to 60.3% at the national level). The population density of 50 inhabitants per km^2^ is slightly higher than the national average (Monographie régionale souss massa, 2020) (Figure 1).

### Flora biodiversity of Souss Massa region

**FIGURE 2:**
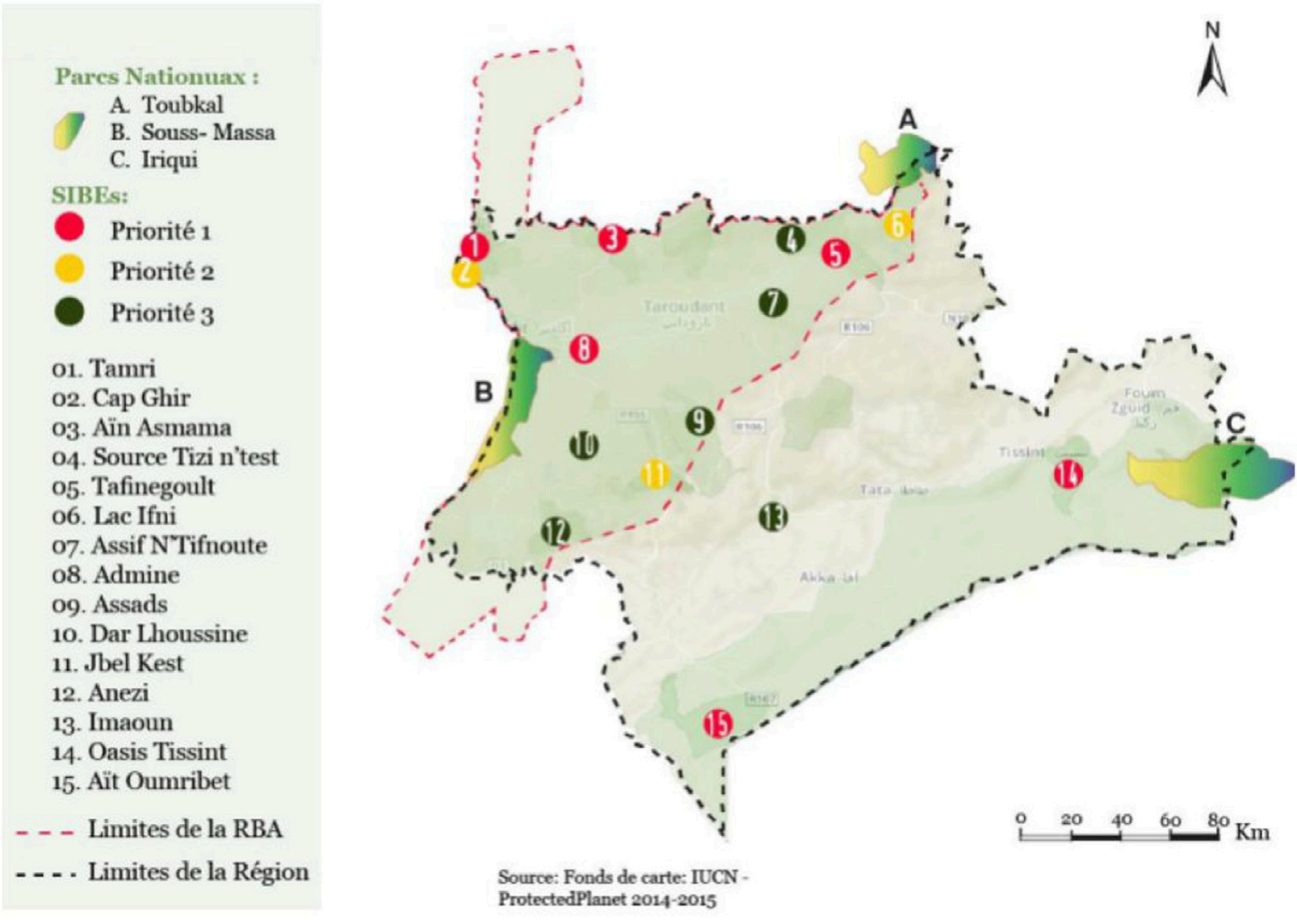
Map of the Sous Massa Region and flora diversity of the region; Ministry Of Energy Transition And Sustainable Development, Morocco (SIREDD, 2025).

The Souss Massa region is a vast fertile plain punctuated on the edge of two river valleys. This bioclimatic zone is of botanical and environmental interest. The coexistence of both Mediterranean and Saharan regions in this area allows it to have species originating from both climates. Some of these species are rare or even threatened, and they do not extend beyond this natural border (Author anonymous, 2025a). Due to specific environmental conditions, the flora has had to adapt, evolve, and over time, differentiate from the rest. The region is home to around fifty endemic plant species (Author anonymous, 2025b). On a territorial scale, the total forest area is estimated at over 1.5 million hectares, including Argan trees, Junipers, Pine, and Evergreen Oak. Approximately 801,810 ha are prominently occupied by the Argan tree in a more or less pure state, with various combinations with other forest species or on cultivated lands as the main species (SIREDD, 2025) (Figure 2).

### Study design and period

This is a cross-sectional, descriptive, and analytical study of women attending the Obstetrics and Gynecology Service at the regional hospital center Hassan II conducted in the region of Souss Massa. This study was conducted from April 2022 to December 2022 to assess the consumption of herbal medicines among pregnant and postpartum women.

### Sample size determination and sampling method

Using the prevalence of a previous study (Ekrasarian et al., 2017). The sample size was determined by using the single-proportion formula.

$$n=\frac{Z^{2}.p.\left(1-p\right)}{E^{2}}$$

Based on the assumption that 10% of participants would not reply and the prevalence is 48% a conservative choice (p = 0.05) has been employed. To ensure that the estimation error would be at most alpha 0.05 with 95% confidence. The calculated optimal sample size was 383. Three hundred eighty-three pregnant women formed the initial calculated sample size, but the sample size was expanded to 420 to account for any missed data or non-response rate to ensure reliability. A convenient sampling technique was used in this study (Bartlett et al., 2025; Cochran, 1977). In this survey, we employed random sampling. Each day, we visit the gynecology and obstetrics department to record new patient cases. We then compile a database of all pregnant and postpartum women who have visited the department in the past 24 h. This list is entered into Excel, and using the random function, we randomly select participants from the list.

### Study population

The study chose to include all of the expecting pregnant women who came forward to the Obstetrics and Gynecology Service at the regional hospital center Hassan II either for the prenatal consultation (PNC) or for giving birth. To collect as much as possible data on the use of herbal medicines during pregnancy and childbirth, the research systematically enrolled all women who were admitted to the hospital’s maternity department during the study period.

Inclusion criteria: all voluntary and consenting pregnant or post-partum women presenting themselves for prenatal consultation at all of the primary healthcare institutions in the network, and women who were admitted to give birth at the maternity at the regional hospital center Hassan II.

### Questionnaire

The questionnaire was prepared and reviewed by a panel of specialists in the fields of clinical pharmacology and biostatistics for content validity. A primary survey was used to conduct a pilot study. The pilot study aimed to measure the respondents’ comprehension of the questions. There were five sections in the questionnaire. The first component of the survey asked questions about the respondents’ socioeconomic status and demographic details, including their age, place of residence, level of education., income, occupation, and social security. The second section is related to pregnancy such as exercise, diet, smoking, and alcohol intake. It includes also the reason for consultation and the time of pregnancy. The third part of the questionnaire covered information on the use of herbal medicine, including the common names for each species, the methods of preparation and administration, the period of use and reasons for use, and their sources of information. The last part includes some questions about the safety of medicinal herbs used by the participants. Women who were pregnant in their first trimester provided information on self-medication during this period, those who were pregnant in their second and third trimesters released details on the first and second trimesters, and those who were pregnant in their third and final trimesters or were in term of childbirth provided information on the whole period of their pregnancy. The questionnaire is attached to the manuscript as an appendix for your reference.

### Data collection procedure

Women who met the study’s eligibility requirements and consented were asked to complete the questionnaires on their own. However, the researchers conducted oral interviews with a small number of illiterate women to complete their questionnaires. The researchers avoided lead questions to prevent any biases and just read the questions for those women. When necessary, the women received explanations to make sure they comprehended the questions.

The research administrators addressed the subjects and explained the purpose of the study to them. All participants were then given an informed consent form to sign before receiving a printed copy of the surveys and were given time to complete them. The completed questionnaires were securely stored in a locked cabinet to ensure confidentiality. Access was restricted to authorized personnel only, and electronic copies were stored on a password-protected computer system to further safeguard the data. The ethnobotanical data was collected, as well as the botanical name of the plant, family name, vernacular name, plant part(s) used in herbal preparation, mode of application to treat women’s diseases, and the method(s) by which it is prepared (i.e. decoction, maceration, power, or infusion …). Data was uploaded and saved into a well-structured and organized file that ensures data accuracy, consistency, and ease of analysis.

### Ethical approval

The biomedical research ethics committee of the Faculty of Medicine and Pharmacy in Rabat, Morocco approved the study protocol. Before starting, the study was approved by the biomedical research ethics committee at Mohamed V University (68-21). All procedures were performed by the revised Declaration of Helsinki published in October 2013 (WMA, 2025). Additionally, a signed informed consent form was provided from each participant. These were worded and indicated the purpose of the study. Confidentiality was ensured by giving each participant a code number for analysis. There was no financial assistance, no rewards, and no conflict of interest. Each responder had the option of not taking part in the survey. It was also emphasized that their access to medical treatment will not be impacted.

### Statistical analysis

For the study subjects, descriptive statistical analyses were conducted. The mean and standard deviation (SD) were used to describe continuous variables, while proportions were used to summarize categorical variables. Demographic and pregnancy-related factors were evaluated and compared with regard to herbal medicine usage. The chi-square test was used to compare data. Statistical significance was considered at P < 0.05 (Author anonymous, 2025b; Jamovi, 2025).

### Relative frequency of citation (RFC)

Ethnobotanical data were analyzed using the frequency of citation (FC) and relative frequency citation (RFC) to determine which species were well-known and most used by the participants in this study. The frequency of citation (FC), or the number of informants who reported using a species, was divided by the total number of informants who took part in the survey (N), to get the relative frequency of citation (RFC). RFC index varies from zero (no one mentions the plants as valuable) to one (every informant would consider it as useful) (Tardío and Pardo-de-Santayana, 2025).

RFC=FCN 0<RFC<1.

## Results

### General characteristics of the population surveyed

The characteristics of the study participants are shown in Table 1. A total of 420 pregnant women and postpartum women were consecutively interviewed. The mean age was 28.7 ± 6.35. Almost all (95.7%) of the respondents were married, 51.4% were living in rural areas, 87.9% were housewives, 42.1% had at least a middle school education and only those with a university level represented 4%. More than half of the participants (72.1%) belonged to low-income households. Women with health insurance made up 23.1% of the population, while those insured by the Medical Assistance Scheme for Economically Underprivileged Persons (RAMED),Medical Assistance Scheme, represented 24%. In terms of gestation, 67.4% were postpartum, and 22.9% third-trimester.

**TABLE 1:** Socio-demographic and obstetric characteristics of participants (*n* = 420) and their association with Herbal medicine use during pregnancy.

Variable	Overall n (%)	Non Herbal Medicine users n (%)	Herbal Medicine users n (%)	P-value
Age				
	28.7 ± 6.35	28.8 ± 6.15	28.6 ± 6.57	0.700
Family situation				
Not Married	18 (4.3)	7 (3.2)	11 (5.4)	0.259
Married	402 (95.7)	211 (96.8)	191 (94.6)	
Origin				
Rural	216 (51.4)	118 (54.1)	98 (48.5)	0.250
Urbain	204 (48.6)	100 (45.9)	104 (51.5)	
Nationality				
Not Moroccan	3 (0.7)	2 (0.9)	1 (0.5)	1.000
Moroccan	417 (99.3)	216 (99.1)	201 (99.5)	
Education				
Illiterate	133 (31.7)	62 (28.4)	71 (35.1)	0.140
Educated	287 (68.3)	156 (71.6)	131 (64.9)	
Employement				
Unemployed	369 (87.9)	191 (87.6)	178 (88.1)	0.874
Employed	51 (12.1)	27 (12.4)	24 (11.9)	
Income level				
Low	303 (72.1)	161 (73.9)	142 (70.3)	0.417
Middle	117 (27.9)	57 (26.1)	60 (29.7)	
Medical insurance				
None	222 (52.9)	117 (53.7)	105 (52)	0.729
Insured	198 (47.1)	101 (46.3)	97 (48)	
Parity				
Nulliparity	44 (10.5)	25 (11.5)	19 (9.4)	0.491
Parity	376 (89.5)	193 (88.5)	183 (90.6)	
Nutrition				
Healthy	312 (74.3)	168 (77.1)	144 (71.3)	0.176
Mixed	108 (25.7)	50 (22.9)	58 (28.7)	
Sport				
No	229 (54.5)	112 (51.4)	117 (57.9)	0.178
Yes	191 (45.5)	106 (48.6)	85 (42.1)	
Yes	1 (0.2)	0 (0)	1 (0.5)	
Pregnancy Age				
Pregnant	125 (29.8)	61 (28)	64 (31.7)	0.407
Postpartum	295 (70.2)	157 (72)	138 (68.3)	
Malformation				
No	416 (99)	218 (100)	198 (98)	0.053*
Yes	4 (1)	0 (0)	4 (2)	
Premature				
None	416 (99)	216 (99.1)	200 (99)	1.000
Yes	4 (1)	2 (0.9)	2 (1)	
Miscarriage				
None	405 (96.4)	213 (97.7)	192 (95)	0.143
Yes	15 (3.6)	5 (2.3)	10 (5)	
Gestational diabetes				
None	346 (82.4)	178 (81.7)	168 (83.2)	0.683
Yes	74 (17.6)	40 (18.3)	34 (16.8)	
Hypertension				
None	383 (91.2)	201 (92.2)	182 (90.1)	0.447
Yes	37 (8.8)	17 (7.8)	20 (9.9)	
Pregnancy at Risk				
None	127 (30.7)	61 (28)	68 (33.7)	0.207
Yes	291 (69.3)	157 (72)	134 (66.3)	
Pregnancy Follow up				
Not followed	125 (29.8)	61 (28)	64 (31.4)	
Followed	295 (70.2)	157 (72)	138 (68.3)	
What type of treatment do you prefer to take during pregnancy				
None	40 (10.7)	27 (13.4)	13 (7.6)	0.020*
Herbal Medicine	82 (22)	37 (18.4)	45 (26.2)	
Drugs	217 (58.2)	124 (61.7)	93 (54.1)	
Both	34 (9.1)	13 (6.5)	21 (12.2)	

^∗^ Values in bold represent statistically significant differences (p < 0.05)

Designing the habits during pregnancy, the majority of the respondents 74.3% have a healthy diet, generally, they have a Mediterranean diet. 45.5% of women are physically active.

### Prevalence of herbal medicine consumption among pregnant and postpartum women

The prevalence of herbal medicine consumption among pregnant and postpartum women is 48% with Confidence interval CI 95% (43.2%–52.7%). A total of 202 women who participated in the survey reported using an herbal medicine during pregnancy. A comparison of the Socio-demographic and obstetric characteristics of herbal and non-herbal medicine users during pregnancy. The results indicated that no significant difference (p-value >0.05) resulted in all sociodemographic and obstetric characteristics, with the exception in terms of malformation and the type of treatment that the participants prefer to take during pregnancy which showed a significant difference p-value <0.05 (Table 1). As revealed in Table 1, pregnant and postpartum women who reported a malformation used herbal medicine (p-value = 0.053) and 26.2% of women who preferred to be treated by herbal medicine used it during pregnancy and 61.7% of women who preferred to use drugs reported that they did not use the herbal medicine during pregnancy (p-value = 0.020).

### Diversity of plant species used

According to the survey, 35 varieties of medicinal plants from 22 families were used during pregnancy to treat gynecologic and obstetrics diseases. A list of the scientific and common names, the family, illness treated, plant part(s), method of preparation, and mode of administration are presented in Table 2. The Lamiaceae and Apiaceae families contributed the most species with (Pallivalappila et al., 2013), followed by Compositae (Adams et al., 2009), and Alliaceae (World Health Organization, 2002). Each of the other families had one species. However, the current study results reported that mugwort *(Artemisia herba-alba* Asso*),* lavender *(Lavandula angustifolia* Mill.), thyme (*Thymus maroccanus* Ball*),* fennel (*Foeniculum vulgare* Mill.)*),* fenugreek *(Trigonella foenum-graecum* L)*,* verbena *(Aloysia citriodora* Palau) and cinnamon (*Cinnamomum verum* J. Presl.) are the most plants that are cited by the pregnant women with FC of 43, 33, 28,25, 19, and 11respectively as presented in Table 2.

**TABLE 2:** List of herbal medicine used among pregnant and postpartum women.

Plant family	Plant species	Vernacular name	Preparation method	Solvant used	Used parts	Administration route	Reason of use	Frequency of citation Fc	Relative frequency of citation RFC
Alliaceae	*Allium cepa* L.	Onion	Decoction	Milk	Fruit, Seeds	Oral	Cold/Flu/Cough Allergy	2	0.005
	Allium sativum L.	Garlic	Decoction	Olive Oil	Fruit	Oral	Respiratory diseases	1	0.002
Apiaceae	Cuminum cyminum L.	Cumin	Infusion	Water	Seeds	Oral	Abdominal Pain Intestinal Gas.	1	0.002
	*Pimpinella anisum* L.	Green anise	Powder, maceration, infusion	Water	Seeds	Oral	Gastric pain Abdominal Pain Intestinal Gas. Cough	5	0.012
	*Foeniculum vulgare* Mill.	Fennel	infusion, decoction, Powder	Water	Seeds	Oral	Abdominal Pain Intestinal Gas. Nausea and Vomissement Gastric pain	28	0.067
	*Ammodaucus leucotrichus Coss*. Durieu	Woolly cumin	Infusion	Water	Seeds	Oral	Abdominal Pain Intestinal Gas.	1	0.002
	*Carum carvi* L.	Caraway	Infusion	Water	Seeds	Oral	Intestinal Gas	1	0.002
Brassicaceae	*Lepidium sativum* L.	Cress Garden	Infusion, decoction	Milk, Eggs Yolk	Seeds	Oral	Amenorrhea. Accelerate labor (Childbirth)	6	0.014
Compositae	*Artemisia herba-alba* Asso	Mugwort	Infusion	Water,Tea	Whole Plant	Oral/Vaginal	Induces an abortion Abdominal pain. pruritus, vaginal itching. Urinary infections Gastric Pain	43	0.102
	*Dolomiaea baltalensis* Dar & Naqshi	Costus Indien	Infusion	Water	Roots	Oral	Diabetes	1	0.002
	*Matricaria chamomilla L*.	Chamomile	Infusion	Water	Flowers	Oral	to relieve and reduce stress. Abdominal and uterine pain	4	0.010
Fabaceae	*Trigonella foenum-graecum* L.	Feneugrec	Maceration, powder	Water, Honey	seeds	Oral	Epigastralgie, Pyrosis To induces an Abortion Appetite, To increase weight	25	0.060
Fagaceae	*Quercus lusitanica* Lam.	Gall Oak	Decoction	Water	fruit	Vaginal	Vaginal itching Pruritus	1	0.002
Iridaceae	*Crocus sativus* L.	Saffron	Infusion	Water,Tea	Flower stigma	Oral	Acceleration of Labor Cold/Flu/Cough	6	0.014
Lamiaceae	*Lavandula angustifolia* Mill.	Lavender	Infusion,fumigation	Water	Leaf	Oral/Vaginal	Vaginal itching induces an abortion. Infection urinary Flu/cold. Diabetes	33	0.079
	*Rosmarinus officinalis* L.	Rosemary	Infusion, decoction	Water		Oral	Intestinal Gas	5	0.012
	*Thymus maroccanus* Ball	Thyme	Infusion,decoction, fumigation	Water, Milk, Tea	Leaf	Oral/Vaginal/Nasal	Cold/Flu/Cough, Urinary burns, Abdominal pain, Asthma Diabetes, Facilte Labor (Childbirth), Epigastralgie, Pyrosis. Amenorrhea Intestinal Gas pruritus, vaginal itching	33	0.079
	*Marrubium vulgare* L.	Marrube blanc	Infusion, decoction	Water	Whole Plant	Vaginal	Vaginal itching	2	0.005
	*Salvia officinalis* L.	Sage	Infusion	Water,Tea	Leaf	Oral	Diabetes, Fertility	5	0.012
Lauraceae	*Cinnamomum verum* J. Presl	Cinnamon	Powder	Milk, Cola, Eggs Yolk, Orange Juice	Internal bark of the tree	Oral	induces an abortion Gastric Pain, Amenorrhea, Anemia, Accelerate labor	11	0.026
Leguminosae	*Glycyrrhiza glabra* L.	Licorice	Infusion, decoction	Water	Roots	Oral	Allergy, To Induce an Abortion	2	0.005
Lythraceae	*Punica granatum* L.	Grenade	Infusion	Water	Fruit peels	Oral	Nausea, Vomissement	1	0.002
Myrtaceae	*Syzygium aromaticum* (L.) Merr. and Perry	Clove	Infusion,fumigation	Water	Seeds	Oral, Nasal	Cold/Flu/Cough	1	0.002
Oleaceae	*Olea europaea* L.	Olive	Extraction	-	Oil	Dermal, Oral	Vaginal itching Flu/cold/Cough	3	0.007
Pedaliaceae	*Sesamum indicum* L.	Sesame	Maceration	Water	Seeds	Oral	Gastric pain	1	0.002
Ranunculaceae	*Nigella sativa* L.	Nigella	infusion, powder	Water, Honey, Olive Oil	Seeds	Oral	Abdominal and uterine COVID-19. To Induce Labor	7	0.017
Rosaceae	*Prunus dulcis* (Mill.) D.A.Webb	Almond	Extraction	-	Oil	Dermal	Stretch marks	1	0.002
Rubiaceae	*Rubia tinctorum* L.	Rubia	Powder	Honey	Whole Plant	Oral	Anemia	1	0.002
Schisandraceae	*Illicium verum* Hook.f.	Star anise	Infusion	Water	seeds	Oral	Cold/Flu/Cough Allergy Respiratory diseases	2	0.005
Verbenaceae	*Aloysia citriodora* Palau	Verbena	Infusion, decoction	Water,Tea	Leaf	Oral	Abdominal and uterine pain Intestin gas Hight blood pressure to relieve and reduce stress	19	0.045
Xanthorrhoeaceae	*Aloe vera* (L.) Burm.f.	Aloe	Infusion	Water	Leaf	Oral	to relieve	1	0.002
Zingiberaceae	*Zingiber officinale* Roscoe	Ginger	Infusion, decoction	Water, Milk	Roots	Oral	Cold/Flu/Cough to induces an Abortion	3	0.007
Zygophyllaceae	*Peganum harmala* L.	Harmel	Fumigation	Water	Seeds	Oral/Nasal	To induce Abortion Cold/flu/Cough	3	0.007

### Aliments treated using medicinal plant species

Medicinal plant species were used to treat a total of 22 diseases. The diseases related to the period of pregnancy and delivery like digestive disorders, infections, anemia, and finally skin complications, sleep disorders, and weight loss are the final three less common causes we have noticed among some users. Several illnesses were treated with a single plant species, while others were treated with a combination of plant parts from various species. In contrast to herbal mixes, monotherapy preparations were more common. Table 2.

### Method of preparation and administration

The current findings revealed that herbal preparations are administered through a variety of routes. The methods of preparation are shown in Table 2. Plant extracts were primarily prepared through decoction, infusion, maceration, Fumigation, extraction, and powder. The main solvent used in the preparation of herbal remedies was water and milk. Some of the preparation was prepared using specific ingredients such as orange juice, egg yolk, and a tea infusion. Infusions (69.69%) were frequently employed, which involves soaking plant material in hot or warm water and letting the combination cool. Decoctions (33.3%) were prepared by boiling plant materials in a specific amount of water for 15–20 min and allowing the mixture to cool before administration. In 9.9% of the plant species, maceration was employed to extract a liquid for patient administration by crushing plant components from a single species or a mixture of plant parts from several species. Minor preparation methods such as pounding (18.18%) like usage of the powder of the plants and fumigation were used with a low frequency in the range of 12.12% % of the medicinal plant species. Fumigation of the plant was used by dropping a mixture of plants in a bowl filled with boiling water, the mode of use is to inhale the steam while holding a towel over the nose. There is another Moroccan traditional method of fumigation consisting to use hot charcoal to burn the plant so that the smoker is sprayed by the user. Extraction (6.06%) is defined as the separation of triglyceride (TAG) using a variety of mechanical or chemical manipulation techniques.

The oral route was the most common route of administration used about the number of plates used by pregnant and postpartum women (90.9%), followed by vaginal (15.15%), nasal (9.9%), and dermal (6.6%). The oral route was administered by swallowing dry powdered plant materials, particularly plant seeds, drinking decoctions, infusions, and maceration extracts (Table 2).

### Reasons and source of information for herbal medicine usage during pregnancy

As shown in Table 3, 92.9% of participants agreed that herbal medicine uses are not harmful to pregnant women and for the fetus and it can be used freely during pregnancy. Although 22% of the participants use herbal medicine because they are available and more accessible compared to medical therapy. Only 3 (2.4%) of the participants reported that herbal medicine costs are cheaper than medical therapy. Seventy (52.6%) of the respondents reported using herbal medicine based on their previous experiences and 44.4% of the participants were recommended by their family, and 3.8% by friends and physicians, respectively. Only 2.3% of the participants get information from the internet about the use of herbal medicine.

**TABLE 3:** Reasons and sources of information about herbal medicine uses by the participants during pregnancy.

Reasons of herbal medicine uses	n (%)^a^
They are available and more accessible compared to medical therapy	28 (22)
Their cost is cheaper than medical therapy	3 (2.4)
They are not harmful for you and your baby during pregnancy.	118 (92.9)

Sources of information	n (%)^a^
Family	59 (44.4)
Friends	5 (3.8)
Internet	3 (2.3)
Myself	70 (52.6)
Physician	5 (3.8)

^a^ Total percentages exceed 100% due to the selection of more than one source.

### Disclosure, satisfaction, and side effects of herbal medicine use in pregnancy

Only 11.2% of the participants were asked if they disclosed herbal medicine used to a medical doctor. The majority of the participants (78.1%) reported that they remark on the benefits of taking herbal medicine during pregnancy. In contrast, some of the participants (5.9%, 4.4%), respectively, observed special side effects and suspected a miscarriage from taking herbal medicine during pregnancy (Supplementary Table S1).

## Discussion

This is the first ethnopharmacology study conducted among pregnant and postpartum women. This study aims to determine the prevalence, and motives behind herbal medicine consumption during pregnancy, during labor, and after delivery in the Hospital Center Hassan II at Souss Massa region. The findings of this study showed that the prevalence of herbal medicine consumption among pregnant and postpartum women at Hospital Center Hassan II of Souss Massa region was 48% with CI 95% (43.2%–52.7%). This is identical to the prevalence found in a similar study. 48.6% reported in a study conducted at the University of Gondar teaching hospital (northwest Ethiopia) (Mekuria et al., 2017), 40% in Palestine (Al-Ramahi et al., 2013) and 48.4% (Abdollahi et al., 2018), 48% (Ekrasarian et al., 2017) each in Iran. In Morocco, a study showed that pregnant women in the province of Guelmim also use MPs during pregnancy with a prevalence of 66.96% (Kamel et al., 2023). Similar findings are reported in other studies with the prevalence of medicinal plants being very high (Eid et al., 2020; Ali-Shtayeh et al., 2015; Yazdi et al., 2019; Dabirifard et al., 2017). This rate is significantly high compared to our findings. These variations in prevalence could be associated with the existence and implementation of regulations governing the sales of medicinal plants, the affordability and availability of herbal products, as well as cultural variations among study participants which differ from one country to another.

This study showed that the participants reported that the main reason for medicinal plant use are easy accessibility, effectiveness, lower cost and there are not harmful for the pregnant woman and for her baby. This finding is consistent with other studies conducted at Hossana town health facilities (Bayisa et al., 2014), and a review of published literature from Middle Eastern countries all of which indicate similar reasoning for herbal medicine use (John and Shantakumari, 2015).

The present study showed that the most common use of herbal medicine among pregnant women was mugwort *(Artemisia herba-alba* Asso*),* lavender *(Lavandula angustifolia* Mill.), thyme (*Thymus maroccanus* Ball*),* fennel (*Foeniculum vulgare* Mill.)), fenugreek *(Trigonella foenum-graecum* L*),* verbena *(Aloysia citriodora* Palau) and cinnamon (*Cinnamomum verum* J. Presl.). In a study conducted in a region located in the south of Morocco, they found that the herbs used were (mugwsort) *A. herba-alba Asso*, (thyme) *T. maroccanus* Ball., (verbena) A*. citriodora Palau*, and*,* (fenugreek) T*. foenum-graecum* L. This similarity can be explained by the identical geographic culture between the two regions: Sous Massa and Guelmim wish both are located in the south of Morocco. In this region, there is a large number of species which have been traditionally used by local communities for generations. The knowledge about these natural products is deeply rooted in the local culture and is largely based on empirical practices and oral transmission (Saadi et al., 2013). Many of these plants have documented uses in ethnopharmacology, demonstrating a strong link between traditional knowledge and scientific findings. Therefore, the natural products identified in this study align with previous ethnopharmacological knowledge, highlighting the importance of preserving and further exploring these traditional practices (Barkaoui et al., 2022; Barkaoui et al., 2017).

In the other country, there is a large number of species used by pregnant women. To manage diseases related to pregnancy. For example, Asteraceae, Lamiaceae, and Solanaceae were dominant families in a study conducted in Uganda (Tugume et al., 2016; Tugume and Nyakoojo, 2019). This large use is due to the vast amount of bioactive compounds found in them (Thomas et al., 2009). Other results at Dessie Referral Hospital, Northeast Ethiopia showed that the medicinal herbs used during pregnancy were *Zingiber officinale* Roscoe, *Allium sativum*, Ocimum lamiifolium Hochst and *Ruta chalepensis* (Belayneh et al., 2022). Those examples are from two countries in Africa but they are different. In a systematic review conducted in sub-Saharan Africa, the top herbal medicines cited in the studies were *Zingiber officinale* Roscoe, *Allium sativum* L*, Cucurbita pepo* L.*, Ricinus communis* L.*, Vernonia amygdalina* debile *and Garcinia kola* heckel was the most common species for the treatment of pregnancy-induced nausea and vomiting and reported in 15 studies (El Hajj and Holst, 2020). While the frequently used herbs in most studies from the Euro-Mediterranean region are peppermint, (ginger) *Zingiber officinale* Roscoe, (thyme) *Thymus maroccanus* Ball, (chamomile) *Matricaria chamomilla* L., (sage) *Salvia officinalis* L., (anise) *Pimpinella anisum* L., (fenugreek) *Trigonella foenum-graecum* L., and (green tea) *Camellia sinensis* (John and Shantakumari, 2015).

The current findings revealed that herbal preparations are administered through a variety of routes. The oral route was the most common route of administration, followed by vaginal, nasal, and dermal routes. Similar findings are reported in other studies (Kamel et al., 2022; Al-Ramahi et al., 2013; Ali-Shtayeh et al., 2015; Raoufinejad et al., 2020). Oral administration often leads to the systemic absorption of active compounds, which can pose greater risks to the pregnancy due to the potential for higher bioavailability and systemic circulation, especially when the compounds cross the placenta. On the other hand, dermal administration typically results in local absorption, potentially limiting the systemic exposure and thus reducing the risk to the fetus. The absorption of active compounds through the skin is often lower, which may lead to a decreased likelihood of adverse effects compared to oral administration. However, the dermal route is not without risks, as certain compounds could still be absorbed into the bloodstream through the skin, albeit at lower levels (Sarecka-Hujar and Szulc-Musioł, 2025). Previous ethnopharmacological knowledge suggests that many traditional practices favor topical applications of herbal products, believing them to be safer for pregnant women. However, more research is required to further explore these differences in terms of both clinical safety and efficacy, particularly with regard to the specific herbs used during pregnancy. Therefore, a careful distinction must be made between the risks associated with oral and dermal herbal remedies, and their potential effects on maternal and fetal health should be considered accordingly (Ahmed et al., 2022).

Plant extracts were primarily prepared through decoction, infusion, maceration, Fumigation, extraction, and powder. The main solvent used in the preparation of herbal therapies was water and milk. This is consistent with other studies conducted in Morocco and elsewhere in the world (Kamel et al., 2022; Eid et al., 2020; Sadeghia and Mahmood, 2014; Ali-Shtayeh et al., 2015; Nergard et al., 2015).

Herbal medicine was used to treat diseases that are related to the period of pregnancy and delivery like digestive disorders, infections, anemia, and skin problems. Several illnesses were treated with a single plant species, while others were treated with a combination of plant parts from various species. Whereas other studies reported this use for stimulating labor or facilitating labor and delivery (Raoufinejad et al., 2020; Tabatabaee, 2011; Soleymani and Makvandi, 2018). Other uses were specifically to enhance neonates’ intelligence and to promote fetal health (Ekrasarian et al., 2017).

The quality and source of information received on herbal medicine influence automatically in the choice of treatment for maternal illnesses. In the research, the most frequent sources of referrals and information were family and friends. This finding is identical to other studies (Abdollahi and Yazdani Chareti, 2019; Al Essa et al., 2019; Kamel, 2013; and Quzmar et al., 2021). Other participants reported physicians as a source of information. Healthcare professionals were also mentioned as a source of information in the studies reviewed. In a Russia, physician recommendations were most often cited (Kennedy et al., 2016). In other places such as a Norwegian study, 80.7% of doctors rated their awareness of herbal medicine as poor (Thomas and Coleman, 2004). As a result, in order to effectively manage herbs during pregnancy, healthcare providers should update their knowledge of the effectiveness, potential hazards, side effects, herb-drug interactions, and fundamental ideas. They should also check their patients for the use of herbal medicine (Duraz and Khan, 2011). Only 11.2% of the participants were asked if they disclosed herbal medicine used to a medical doctor. The majority of the participants (78.1%) reported that they remark on the benefits of taking herbal medicine during pregnancy. In contrast, some of the participants (5.9%, 4.4%), respectively, observed special side effects and suspected a miscarriage from taking herbal medicine during pregnancy. The teratogenicity of herbal medications in animal models has to be studied further. Pregnant women should receive health education from prenatal care professionals who are knowledgeable about the data surrounding the possible advantages and hazards of herbal products.

## Conclusion

The high utilization of herbal medicine among pregnant women in the Souss Massa region, as demonstrated in this study, underscores the importance of understanding its widespread use. The majority of participants considered herbal remedies safe during pregnancy, with nearly half perceiving them as more beneficial than conventional medical treatments. This finding highlights a significant area for further research, as it calls for a deeper investigation into both the potential benefits and risks of herbal medicines. This study contributes to expanding our knowledge of the role various plant species play in managing pregnancy-related health issues. However, to fully understand their therapeutic potential, further phytochemical and pharmacological studies are needed to identify their bioactive components. Additionally, to ensure the safe and effective use of these medicines, more research is required to establish standardized dosages and formulations. These efforts will provide clearer guidelines for the use of herbal medicines during pregnancy and help guide future medical research in this field.

## Data Availability

The original contributions presented in the study are included in the article/Supplementary Material; further inquiries can be directed to the corresponding authors.

## Abbreviations

CI, Confidence Interval; FC, Frequence of citation; RAMED, Medical Assistance Scheme (Régime d’Assistance Médicale); RFC, Relative frequency of citation; SD Standard of deviation; TAG, Triglyceride; WHO, World Health Organization.
