# Supplementary material for: An ethnopharmacological survey of herbal medicines use among pregnant and postpartum women in Souss Massa (Morocco)
Source: Front Pharmacol. 2025 May 9;16:1567930. doi: 10.3389/fphar.2025.1567930 (PMC12099448; doi:10.3389/fphar.2025.1567930)
Supplement: Supplementary file 1 [file Table1.docx]

|  | **Number** | **Percent** |
| --- | --- | --- |
| **Disclosure of Herbal Medicine use to a medical doctor** | | |
| Yes | 15 | 11.2 |
| No | 119 | 88.8 |
| **Have you seen any benefits from taking herbal medicine ?** | | |
| Yes | 107 | 78.1 |
| No | 30 | 21.9 |
| **Have you observed any special side effects from the use of herbal medicine ?** | | |
| Yes | 8 | 5.9 |
| No | 128 | 94.1 |
| **Did you suffer a suspected miscarriage during this pregnancy as a result of this use?** | | |
| Yes | 6 | 4.4 |
| No | 129 | 95.6 |

**Supplementary materials**

Suppl. tab 1 : Disclosure, satisfaction and side effects with Herbal medicine uses in pregnancy
